# Development of AS1411 Aptamer-Targeted Solid Lipid Nanoparticles to Improve Anticancer Efficiency of Lawsone

**DOI:** 10.34172/apb.025.45553

**Published:** 2025-11-18

**Authors:** Sara Gheshlaghi, Armita Sedighidarijani, Afsaneh Maali, Maryam Hashemi, Shiva Golmohammadzadeh, Zahra Salmasi

**Affiliations:** ^1^School of Pharmacy, Mashhad University of Medical Sciences, Mashhad, Iran; ^2^Student Research Committee, Mashhad University of Medical Sciences, Mashhad, Iran; ^3^Department of Pharmaceutics, School of Pharmacy, Mashhad University of Medical Sciences, Mashhad, Iran; ^4^Department of Chemical Engineering, Faculty of Engineering, Ferdowsi University of Mashhad, Mashhad, Iran; ^5^Department of Pharmaceutical Biotechnology, School of Pharmacy, Mashhad University of Medical Sciences, Mashhad, Iran; ^6^Nanotechnology Research Center, Pharmaceutical Technology Institute, Mashhad University of Medical Sciences, Mashhad, Iran; ^7^Department of Pharmaceutical Nanotechnology, School of Pharmacy, Mashhad University of Medical Sciences, Mashhad, Iran

**Keywords:** Solid lipid nanoparticle, Lawson, Targeted delivery, Aptamer, Cancer

## Abstract

**Introduction:**

Lawsone (LWS), a naphthoquinone dye known for its anticancer properties, faces challenges with aqueous solubility, which restricts its therapeutic use. Solid lipid nanoparticles (SLNs) are recognized for enhancing the bioavailability of poorly soluble drugs, supporting in targeted drug delivery and reducing toxicity to normal tissues. The purpose of this study was to develop chitosan-coated solid lipid nanoparticles that were loaded with lawsone and conjugated with the AS1411 aptamer (LWS-SLN-Chit-Apt) for evaluating the cytotoxic effects on mouse colon adenocarcinoma cells (C26).

**Methods:**

High-shear homogenization and ultrasound methods were used for producing the LWS-SLNs nanoparticles. Several considerations, including dynamic light scattering (DLS), differential scanning calorimetry (DSC), scanning electron microscopy (SEM), and Fourier-transform infrared spectroscopy (FTIR), were directed to characterize the properties of the nanoparticles. Next, the nanoparticles were coated with chitosan, then conjugated to AS1411 aptamer, which was confirmed with DLS and gel electrophoresis. Additionally, the encapsulation efficiency of LWS and its release profile were investigated. Cytotoxicity and cellular uptake were evaluated on C26 cells and Chinese hamster ovary (CHO) cells.

**Results:**

The LWS-SLNs and LWS-SLN-Chit-Apt formulations revealed particle sizes of 160±14.8 nm and 350±22.5 nm, with encapsulation efficiencies of 70.72±2.64% and 70.00±4.3%, respectively. Both formulations exhibited a sustained drug release profile over 120 hours. Targeted nanoparticles displayed higher cellular uptake and cytotoxicity in nucleolin-positive cells (C26 cells) compared to nucleolin-negative cells (CHO cells), with no substantial differences observed.

**Conclusion:**

These results illustrated that LWS-SLN-Chit-Apt could be considered as a great candidate for further studies and *in vivo* trials.

## Introduction

 Henna (*Lawsonia inermis*) extracts and their bioactive compounds, such as flavonoids, coumarins, catechin, steroids, xanthones, tannins, polyphenols, fatty acids, alkaloids, quinones, leucocyanidin, epicatechin, triterpenoids, and quercetin, have numerous applications across various areas.^1^ It boasts a rich history in traditional medicine, garnering attention for its potential pharmaceutical and medicinal uses.^1,2^ Studies indicate various medical applications, including anti-inflammatory^3,4^, antioxidant^5,6^, antimicrobial^7-9^, anti-tumor ^10^, wound healing ^11^, and its benefits for skin and hair health.^12^

 Lawsone (LWS; 2-Hydroxy-1,4-naphthoquinone), a key colorant constituent of Henna, stands out as one of the most significant members of naphthoquinone-type dyes. LWS and its derivatives have displayed promising efficacy in hindering the growth of various cancerous cells, including ovarian cancer (SKOV-3) ^13^, human lung carcinoma (A549), colorectal cancer (DLD1), and Hepatocellular carcinoma (HepG2).^14^

 Nevertheless, the poor solubility of LWS in aqueous solutions led to some limitations in its bioavailability and medical characteristics. Different nano-delivery systems play an essential role in effective delivery of poorly soluble ingredients, improving their stability, bioavailability, and controlled-release as well as facilitating targeted delivery.^15,16^ A stable LWS-loaded nanoemulsion, prepared using the emulsion phase inversion method, exhibited enhanced cytotoxicity in cervical carcinoma cancer cells in comparison with free LWS.^17^ Furthermore, LWS encapsulated in polylactic-co-glycolic acid nanoparticles (NPs) modified with chitosan and folic acid demonstrated enhanced cytotoxic effects against Panc-1 cells.^18^ Additionally, designed nanoniosomes containing LWS using non-ionic surfactants and cholesterol, displayed sustained release and increased anticancer effects in the MCF-7 cell line compared to free LWS.^19^ Moreover, LWS-SLNs, formulated using the hot homogenization technique, exhibited greater cytotoxicity than free LWS against the A549 cell line and induced apoptosis.^20^ Solid lipid nanoparticles (SLNs) can encapsulate both hydrophilic and hydrophobic compounds, rendering them suitable for delivering LWS, which has a hydrophobic nature. SLNs serve as biocompatible carriers that enhance bioavailability, enable controlled release, and facilitate targeted drug action.^20,21^

 Additionally, some studies suggest coating SLNs with chitosan to enhance stability.^18^ Moreover, incorporating chitosan into SLNs leads to an increase in particle size and improved drug association efficiency.^22^ Chitosan-coated SLNs exhibit significantly delayed drug release compared to uncoated SLNs, indicating a change in the diffusion mechanism.^23^ Furthermore, coating SLNs with positively charged chitosan can change the surface charge of the SLNs, enabling conjugation of negatively charged antibodies and aptamers to the surface of negatively charged SLNs.

 For targeted cancer therapy, aptamers have emerged as promising structures because of their high specificity and capability to bind to specific targets. They can be easily modified and offer advantages such as low molecular weight and non-immunogenicity.^24^ Furthermore, NPs conjugated with aptamers have demonstrated outstanding potential in cancer therapy, offering more efficient and targeted treatment while minimizing toxicity to healthy tissues.^25^ The AS1411 aptamer is a synthetic oligodeoxynucleotide, consisting of 26 nucleotides, with the sequence 5’GGTGGTGGTGGTTGTGGTGGTGGTGG.^26^ The mechanism by which AS1411 aptamer targets cancer cells involves its ability for binding to nucleolin, which is overexpressed on the membranes of these cells, resulting in cytotoxic effects. AS1411 aptamer compete with bcl-2 mRNA for binding to cytoplasmic nucleolin, lead to the destabilization of bcl-2 mRNA and induce tumor cell death.^27,28^ The AS1411 aptamer shows great potential in targeted drug delivery systems by effectively inhibiting proliferation and inducing differentiation in various cancer cells, such as breast cancer cells (MCF-7 and MDA-MB-231^28^), colon cancer cells (SW480 ^29^ and C26 ^30^), ovarian cancer cells (SKOV-3 ^31^), and prostate cancer cells (PC-3^32^). This promising approach offers a considerable improvement in cancer therapy.

 The incorporation of lawsone into solid lipid nanoparticles functionalized with AS1411 aptamer for targeting delivery has not been previously reported in the literature. So, the present study was designed to develop the chitosan-coated solid lipid nanoparticles, which were loaded with lawsone and conjugated with the AS1411 aptamer (LWS-SLN-Chit-Apt). Different physicochemical properties of the nanoparticles were assessed. Drug release profiles, cellular uptake, and the cytotoxic efficacy of the nanoparticles were investigated against the mouse colon adenocarcinoma cell line (C26) and the Chinese hamster ovary cell line (CHO).

## Materials and Methods

###  Chemicals and Reagents

 Murine colon adenocarcinoma cell line (C26) and Chinese hamster ovary cell line (CHO) were obtained from the Pasteur Institute of Iran. Poloxamer 188 and Tween 80 were bought from Uniqema (USA). Sodium Collate, Chitosan, MTT reagent (3-(4,5-dimethylthiazolyl-2)-2,5-diphenyltetrazolium bromide), and dimethyl sulfoxide (DMSO) were purchased from Sigma (Germany). Fetal bovine serum (FBS) and Precirol® were taken from Gibco (USA) and Gattefosse (France), respectively. RPMI 1640 medium, trypsin (0.05 %), and PEN-STREP (100X) were purchased from Bio-idea (Iran). LWS, AS1411 aptamer, Ethanol 96%, and Chloroform, were bought from Gol Exir (Iran), Sinacolon (Iran), JATA (Iran), and Samchun (Korea), respectively. Methanol and Acetic Acid were purchased from Dr.Mojalali (Iran). All the materials used in the study were analytical grade.

###  Preparation of solid lipid nanoparticles containing Lawson (LWS-SLNs)

 The high-shear homogenization and ultrasound method were utilized for the preparation of LWS-SLNs. LWS-SLNs were fabricated using two different solid lipids: glyceryl behenate (Compritol) and glyceryl palmitostearate (Precirol), each at a concentration of 5%. Two types of surfactants were used: Tween 80 as an aqueous phase surfactant and Poloxamer 188 as an oil phase surfactant, along with a mixture of both, at concentrations of 2.5% and 5%. The concentration of LWS was kept constant at 0.1% for all formulations.

 The selection and optimization of lipid and surfactant components were guided by systematic experimental screening and supportive literature evidence. For lipid selection, Precirol®, glyceryl monostearate, and Compritol® were initially screened by assessing the solubility of LWS in melted lipids. Precirol and Compritol provided homogeneous and stable mixtures, while glyceryl monostearate showed phase separation. Between Precirol and Compritol, Precirol’s lower melting point facilitated faster recrystallization and limited crystal growth, yielding nanoparticles with lower polydispersity and more favorable physicochemical stability. Thus, Precirol was chosen as the primary lipid.^33,34^ For surfactant selection, screening of different types and concentrations of surfactants resulted in the combination of Tween 80 and Poloxamer 188 which could enhance the stability and improve the properties of the formulation. This mixture offered superior properties compared to a single surfactant because it prevented particle aggregation and optimized key characteristics like particle size, entrapment efficiency, and Polydispersity Index (PDI), resulting in a more stable and effective formulation.^35-37^ Furthermore, sodium cholate was selected as a co-surfactant to reduce the interfacial tension, prevent the aggregation, and subsequently improve the formulations stability.^38^

 The final concentrations (5% lipid, 2.5% Tween 80, 2.5% Poloxamer 188) were chosen empirically during formulation trials, where these ratios yielded the most stable colloidal dispersion with favorable particle size, PDI, and zeta potential.

 The oil phase consisted of the solid lipid (5%) and LWS (0.1%), as well as the oil phase surfactant. The aqueous phase contained deionized water and the aqueous phase surfactant. Both phases were warmed in a hot water bath (80 ^◦^C) until they dissolved uniformly and reached the same temperature. Subsequently, the aqueous phase was rapidly added to the oil phase and homogenized (5000 rpm) for 5 minutes using a Heidolph homogenizer Diax900 (Schwabach, Germany). The resulting dispersions were subjected to a Prob Sonicator (Soniprep 150, MSE, UK) and sonicated for seven cycles with 60 seconds on and 15 seconds off to prepare nanoparticles.

 To achieve smaller particle sizes of SLNs, 0.5% sodium cholate was added as a co-surfactant to the oil phase of the desired SLNs preparation. Samples were allowed to cool to room temperature overnight.

 LWS-SLNs (500 µL) with deionized water (500 µL) were added into an Amicon® filter (100 KDa pore size) and centrifuged at 5000 rpm, 4°C for 10 minutes, followed by two more washing to eliminate any residual free drugs in the sample.

###  Characterization of LWS-SLNs

 The examination of particle size, polydispersity index (PDI), and surface charge was conducted via dynamic light scattering (DLS) using the Particle size analyzer (Malvern Zetasizer Nano ZS, UK). The physical stability of LWS-SLNs was evaluated with measurement of particle size, PDI, and surface charge after storage periods of 1, 3, and 6 months at temperatures of 4°C, 25°C, and 37°C. The morphology of LWS-SLNs nanoparticles was explored using a scanning electron microscope (SEM). Additionally, the molecular structure and thermotropic behavior of LWS, SLNs, and LWS-SLNs were studied using a Fourier transform infrared spectrometer (FTIR, Nicolet Avatar370) over a wavelength range of 400 cm^−1^ to 4000 cm^−1^ and differential scanning calorimetry (DSC, Mettler Toledo DSC822e), respectively.

###  Coating of LWS-SLNs with Chitosan

 LWS-SLNs were coated with chitosan to make a positive charge on the particles for optimal aptamer binding. Chitosan (10 mg) was dissolved in acetate buffer (10 ml) with a pH of 5 and stirred for 24 hours. Subsequently, LWS-SLNs (375 µL) were added dropwise to this solution (1125 µL) and stirred for 2 hours for coating the negatively charged LWS-SLNs with the positively charged chitosan. The particle size, PDI, and zeta potential of the chitosan-coated LWS-SLNs (LWS-SLNs-Chit) were determined.

###  Binding of AS1411 Aptamer to LWS-SLN-Chit 

 To achieve non-covalent conjugation of the aptamer to NPs, 20 µL of AS1411 aptamer (10 µM) was combined with 180 µL of LWS-SLN-Chit and stirred for 2 hours at room temperature.^39^

###  Confirmation of AS1411 Aptamer Conjugation to the Nanoparticles

 The LWS-SLN-Chit complexes, with or without the aptamer, as well as the aptamer alone, were subjected to electrophoresis on a 2.5% agarose gel in TBE (Tris-Boric Acid-EDTA) buffer to validate complex formation. Electrophoresis was conducted at 80 V in running buffer for 20 minutes and the results were analyzed by alliance 4.7 Gel doc (Uvitec, UK). ^40^ Binding of the aptamer was also evaluated using DLS.^33^ Additionally, the stability of LWS-SLN-Chit-Apt after storage periods of 30 days was evaluated at temperatures of 4°C and 25°C via electrophoresis and DLS assays.^41,42^

###  Evaluation of Drug Encapsulation Efficiency 

 The direct and indirect methods were employed to evaluate the encapsulation efficiency (EE%). In direct method, the remaining SLNs in the apical compartment of the Amicon® filter were suspended in an appropriate methanol: chloroform solvent (2:1) to break down the lipids in NPs structure, then the concentration of LWS after dilution with ethanol was quantified using UV/Vis spectrophotometry (S2100UV, Unico, USA) at a wavelength of 271 nm (λ_max_), with reference to a calibration curve. In the indirect method, the concentration of LWS in the solution that passed through the filter was similarly quantified. Subsequently, the EE% was calculated using equation 1 for the direct method and equation 2 for the indirect method.


Eq.1 $$\text{EE\%}=\;\frac{\text{Weight of Loaded LWS }\left(\text{mgr}\right)}{\text{Weight of Total LWS }\left(\text{mgr}\right)}\;\times 100$$

Eq.2 $$\text{EE\%}=\;\frac{\text{Weight of Total LWS }\left(\text{mgr}\right)-\text{Weight of Unloaded LWS }\left(\text{mgr}\right)}{\text{Weight of Total LWS }\left(\text{mgr}\right)}\times 100$$

###  In Vitro Release

 To estimate the profile release of lawsone, 1 mL nanoparticles (LWS-SLNs and LWS-SLN-Chit-Apt) were individually put into a dialysis bag with a pore size of 12 KDa and dipped in Phosphate-buffered saline (PBS, pH 7.4) and citrate buffer (pH 5.5) containing 0.1% Tween80 as the release medium in a shaker incubator at 37°C and 80 rpm. At different time intervals (0-120 h), the 2 mL released sample was withdrawn from the release tank, and an equal volume of buffer was added back to the tank. The concentration of released lawsone at each sampling interval was quantified by the absorbance measurement at 271 nm (λ_max_), referencing to a calibration curve, using a UV/Vis spectrophotometer. The cumulative percentage of released drug at each sampling point was calculated using equation 3. PBS and citrate buffers containing Tween 80 were used as the blank solutions.


Eq.3 $$\text{Cumulative Drug Release }\left(\text{\%}\right)=\frac{\text{Weight of drug released }\left(\text{mg}\right)}{\text{Weight of drug in the mat }\left(\text{mg}\right)}\times 100$$

###  Cell Culture 

 C26 (mouse colon adenocarcinoma cells), as the nucleolin-expressing cancer cells, and CHO (Chinese hamster ovary cells), as the nucleolin-negative normal cells, were supplied by the Pasteur Institute of Iran. Both cell lines were cultivated in Roswell Park Memorial Institute medium (RPMI-1640) supplemented with 10% fetal bovine serum (FBS), penicillin (100 U/ml), and streptomycin (100 μg/ml). The cells were cultured at 37°C in a humidified atmosphere containing 5% CO­_2_.

###  Cytotoxicity Assay

 The MTT colorimetric assay was utilized to evaluate the cytotoxicity of free LWS, LWS-SLNs, LWS-SLN-chit-Apt, and bare SLNs. In brief, cells were seeded in 96-well plates at a density of 1 × 10^4^ cells per well. The next day, the cells were treated with a range of LWS concentrations (3.125-25 μg/mL) across different formulations. Following 24-hour incubation period, the medium was replaced with fresh medium, and 10 μL (5 mg/mL) of MTT reagent was added to each well, followed by a 4-hour incubation in the dark. The medium was discarded, and subsequently 100 μL of DMSO was added to dissolve the formazan crystals formed by the MTT reagent. Untreated cells served as the control (100% viable). Finally, the absorbance was evaluated using a microplate reader (Biotek Gen5, USA) at 570 and 630 nm wavelengths. ^43^ The percentage of cell viability was determined using equation 4. The IC_50_ represents the concentration of the samples that inhibit cell growth by 50%.


Eq.4 $$\text{\% Cell Viability}=\frac{Abs_{Treatment}-Abs_{Blank}}{Abs_{Control}-Abs_{Blank}}\;\times 100$$

###  Determination of Intracellular Uptake

 C26 and CHO cells were cultured in 24-well plates (5 × 10^4^ cells per well). Following 24hour incubation, different formulations were added to the cells including free LWS, LWS-SLNs, and LWS-SLN-Chit-Apt (containing 10.36 µg/mLof LWS, corresponding to the IC_50_ value for LWS-SLN-Chit-Apt against C26 cells) for 4 hours. The cells were washed three times with PBS buffer to eliminate any extracellular LWS, lysed with DMSO, and harvested by centrifugation. The intracellular LWS concentration was determined using UV/Vis spectrophotometry at a wavelength of 271 nm (λ_max_), with reference to a calibration curve.

###  Statistical Analysis

 Data were presented as the mean ± standard deviation of triplicate measurements. For multiple comparisons, one-way ANOVA followed by Tukey test was applied to find the significant difference between groups. The P value of ≤ 0.05 was considered as a significance level.

## Results

###  Characterization of the LWS-SLNs 

 First, for the preparation of LWS-SLNs, the type of lipid and surfactant, as well as the surfactant percentage, were optimized to obtain the best results for particle size, PDI, and zeta potential. As shown in Table 1, formulations using Precirol as the solid lipid led to smaller particle sizes than those using Compritol. Among the SLNs prepared with Precirol, the formulation containing Poloxamer 188: Tween 80 (%w/v) (1:1) resulted in the smallest particle size and the highest zeta potential (SLN-3 formulation). Finally, to achieve a smaller particle size, sodium cholate was added as a cosurfactant to the oil phase of the SLN-3 formulation. By adding 0.5% sodium cholate as a co-surfactant to the oil phase in this composition, the desired LWS-SLNs formulation was achieved for further analysis, comprising 5% Precirol, 2.5% Poloxamer 188, 2.5% Tween 80, 0.5% sodium cholate, and 0.1% Lawsone. The addition of sodium cholate reduced the particle size of SLN-3 from 259 ± 10.3 nm to 160 ± 14.8 nm and decreased the PDI from 0.224 ± 0.02 to 0.12 ± 0.019, indicating a relatively narrow particle size distribution. The surface charge remained unchanged, with the zeta potential remaining at -20 ± 3.6 mV, compared to -20 ± 3.2 mV.

**Table 1:** Physicochemical characteristics of different formulations

**Formula**	**Solid Lipid (5%w/v)**	**Poloxamer 188: Tween 80 (%w/v)**	**Lawsone**	**Z-average (nm)**	**Zeta potential (mv)**	**PDI**
SLN-1	Precirol	2.5%: 0%	0.1%	Unstable	-	-
SLN-2	Precirol	5%: 0%	0.1%	Unstable	-	-
SLN-3	Precirol	2.5%: 2.5%	0.1%	259 ± 10.3	-20 ± 3.2	0.224 ± 0.02
SLN-4	Precirol	0%: 2.5%	0.1%	421 ± 22.2	-13 ± 1.4	0.3 ± 0.045
SLN-5	Precirol	0%: 5%	0.1%	379 ± 16	-11.3 ± 1.1	0.159 ± 0.019
SLN-6	Compritol	2.5%: 0%	0.1%	Unstable	-	-
SLN-7	Compritol	5%: 0%	0.1%	Unstable	-	-
SLN-8	Compritol	2.5%: 2.5%	0.1%	509.6 ± 29.5	-19.6 ± 3.05	0.282 ± 0.025
SLN-9	Compritol	0%: 2.5%	0.1%	743 ± 32.1	-9.96 ± 1.05	0.271 ± 0.021
SLN-10	Compritol	0%: 5%	0.1%	691 ± 30	-12.4 ± 1.89	0.11 ± 0.012

**Figure 1:**
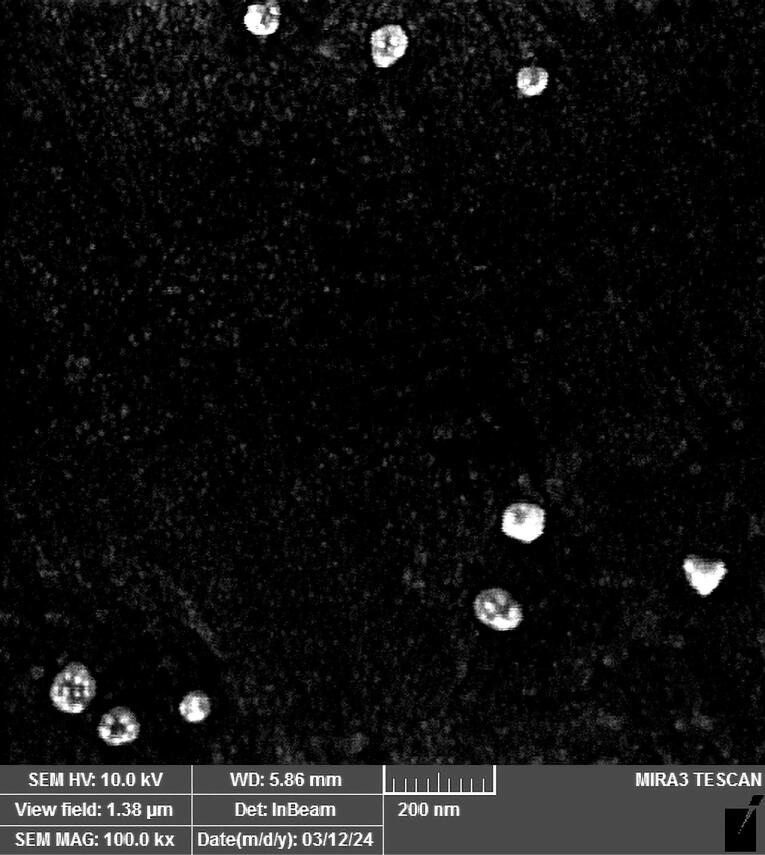
SEM image of LWS-SLNs nanoparticles

 LWS-SLNs were then further examined using SEM to assess their size and morphology. SEM images revealed that the synthesized particles have a smooth and uniform surface, are spherical in shape, and have nano-sized dimensions (Figure 1). In addition, the encapsulation efficiency of LWS-SLNs was found to be 70.72 ± 2.64% and 75.72 ± 2.34% using the direct and indirect methods, respectively.

###  Physical Stability of LWS-SLNs

 The particle size, PDI, and zeta potential of LWS-SLNs after storage for 1, 3, and 6 months at temperatures of 4°C, 25°C, and 37°C were measured and presented in Table 2. At 4°C and 25°C, the particle size did not differ considerably after 1 month of storage; however, at later time points (both 25°C and 4°C), and at all-time points at 37°C, a substantial increase in size occurred compared to fresh LWS-SLNs (*P* < 0.05).

**Table 2:** Physical stability of LWS-SLNs after 1, 3, and 6 months at temperatures of 4°C, 25°C, and 37°C

**Time (Month)**	**Temperature (**°C**)**	**Z-average (nm)**	**Zeta potential (mV)**	**PDI**
Fresh	25	160 ± 14.8	-20 ± 3.6	0.12 ± 0.019
1	4 25 37	171 ± 11.4 183 ± 13.6 324 ± 28.3	-22 ± 2.6 -23 ± 2.5 -28 ± 3.3	0.1 ± 0.01 0.12 ± 0.011 0.12 ± 0.01
3	4 25 37	206 ± 16.1 205 ± 16.5 346 ± 31.2	-20 ± 2.5 -22 ± 3.0 -2.5 ± 3.1	0.2 ± 0.021 0.138 ± 0.02 0.15 ± 0.019
6	4 25 37	210 ± 15.4 206 ± 17.2 368 ± 29.7	-26 ± 3.4 -25 ± 2.9 -31 ± 3.6	0.235 ± 0.022 0.155 ± 0.018 0.185 ± 0.021

###  Differential Scanning Calorimetry (DSC)

**Figure 2:**
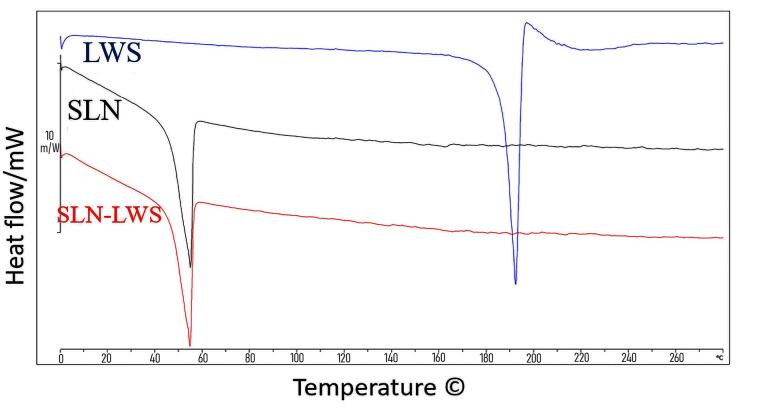
The differential scanning calorimetry (DSC) spectra of SLNs (black), LWS (Blue), and LWS-SLNs (Red)

Figure 2 presents a comparison of the thermograms of the SLNs, LWS, and LWS-SLNs samples during DSC heating. For all lyophilized samples, the DSC thermograms recorded upon heating (from 25 to 280°C) show an intense endothermic peak, indicating the melting point of the freeze-dried powder where the solid transitions to a liquid state. The endothermic peaks were observed at approximately 191.59°C, 54.51°C, and 56°C for LWS, SLNs, and LWS-SLNs, respectively.

###  FTIR Analysis

**Figure 3:**
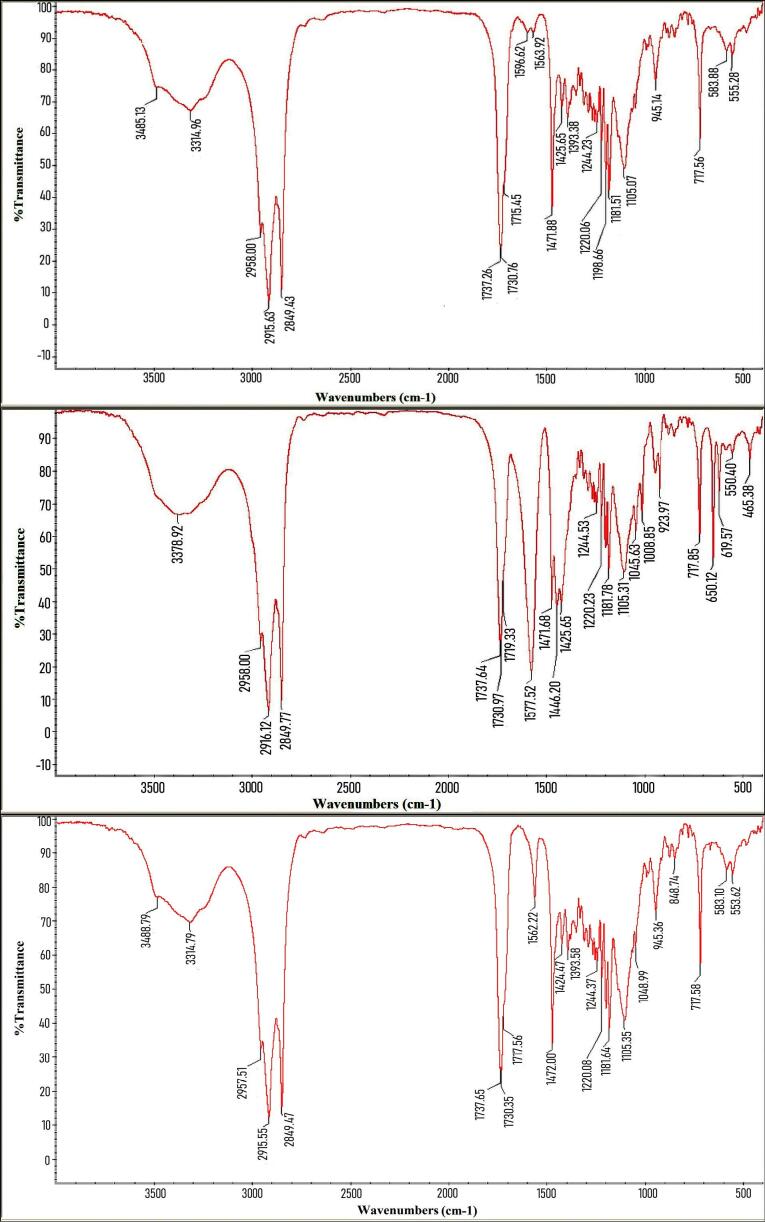
FTIR spectra of a) SLNs, b) LWS, and c) LWS-SLNs

 FTIR analysis was conducted on LWS, SLNs, and LWS-SLNs to investigate the chemical interactions between the components, verify the encapsulation of LWS within the nanoparticles, as illustrated in Figure 3. The results validated the chemical composition of the nanoparticles and the effective incorporation of LWS into the particle matrix.

###  Confirmation of AS1411 Aptamer Binding to LWS-SLN-Chit

 First, the nanoparticles (LWS-SLNs) were coated with chitosan to create a positive charge on the particles for optimal aptamer binding. According to Table 3, after coating with chitosan, the particle size increased from 160 ± 14.8 nm to 304 ± 20.3 nm, and the zeta potential became positive due to the positive charge of chitosan, changing from -20 ± 3.6 mV to + 20 ± 3.2 mV. Subsequently, following the conjugation of the aptamer to LWS-SLN-Chit, the particle size further increased from 304 ± 20.3 nm to 350 ± 22.5 nm. Additionally, due to the negative charge of the aptamer, its binding to LWS-SLN-Chit caused a slight decrease in the zeta potential, from + 20 ± 3.2 mV to + 16 ± 2.1 mV. These changes confirmed AS1411 aptamer binding to the surface of LWS-SLN-Chit.

**Table 3:** Physicochemical properties of formulated SLNs before/after aptamer binding

**Formula**	**Z-average (nm)**	**Zeta potential (mV)**	**PDI**
LWS-SLNs	160 ± 14.8	-20 ± 3.6	0.12 ± 0.019
LWS-SLN-Chit	304 ± 20.3	+ 20 ± 3.2	0.17 ± 0.02
LWS-SLN-Chit-Apt	350 ± 22.5	+ 16 ± 2.1	0.15 ± 0.02

**Figure 4:**
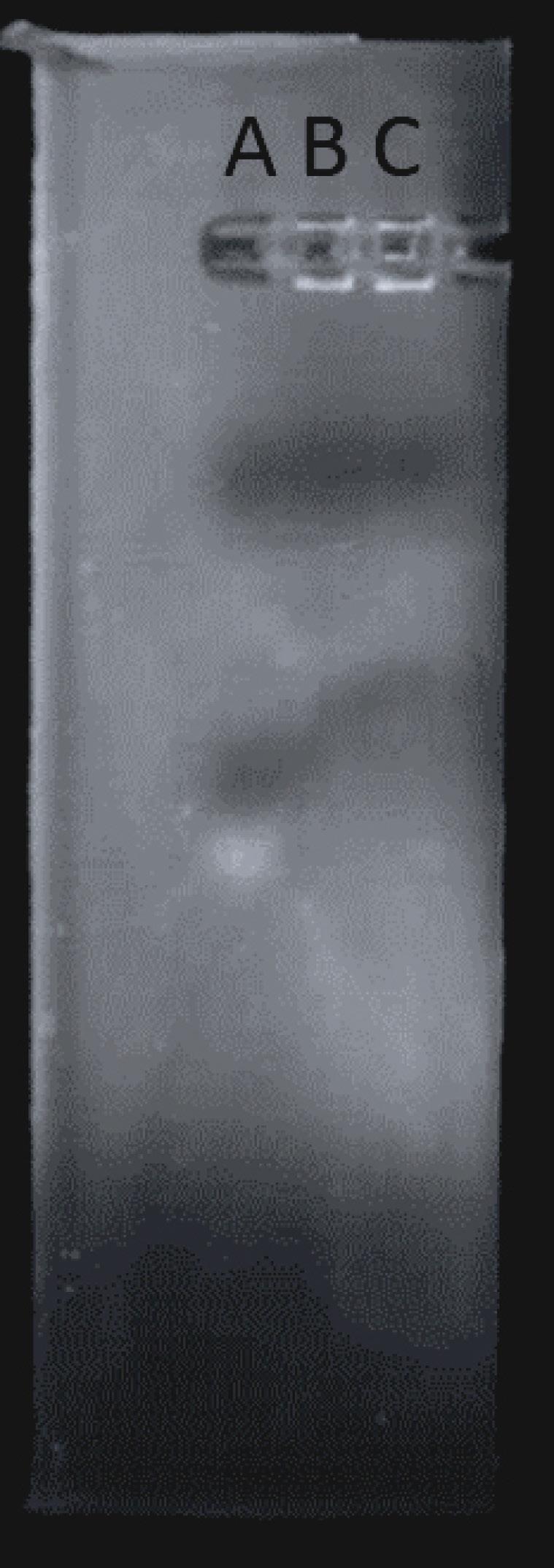
Agarose-gel electrophoresis for confirming the binding of AS1411aptamer to LWS-SLN-Chit. A) Free Apt, B) LWS-SLN-Chit, and C) LWS-SLN-Chit-Apt

 For further verification, the agarose gel electrophoresis was performed. As shown in Figure 4, movement of free aptamer through the gel caused the bright band on the agarose gel (lane A), while LWS-SLN-Chit and LWS-SLN-Chit-Apt remained in the wells attributed to their high molecular weights (lane B and C).

 In addition, the encapsulation efficiency for LWS-SLNs, LWS-SLN-Chit and LWS-SLN-Chit-Apt was 70.72 ± 2.64, 69.64 ± 3.5 and 70 ± 4.3, respectively.

###  Stability of LWS-SLN-Chit-Apt 

**Figure 5:**
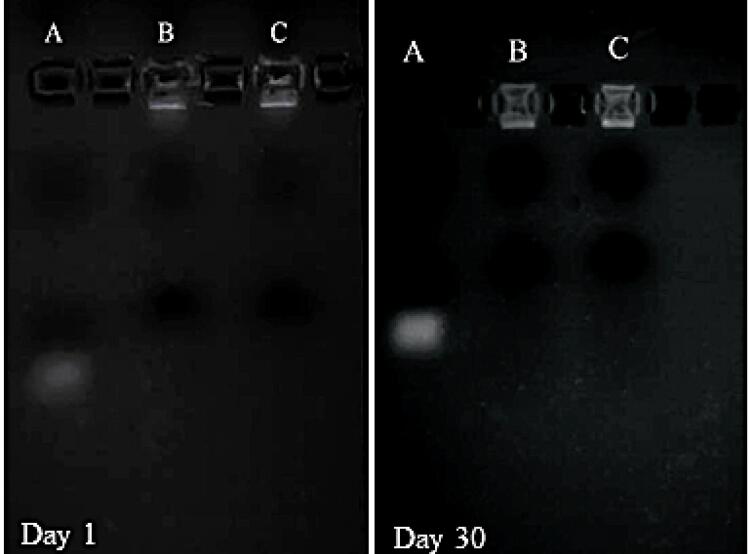
Agarose-gel electrophoresis for confirming the stability of LWS-SLN-Chit-Apt complex after 30 days. A, B, and C in each figure are related to Free Apt, LWS-SLN-Chit-Apt at 4°C, and LWS-SLN-Chit-Apt at 25°C, respectively

 The particle size and zeta potential of LWS-SLN-Chit-Apt after 30 days of storage at 4°C and 25°C are shown in Table 4. The particle size of the complex did not change significantly after 30 days of storage at 4°C. Conversely, the particle size increased gradually at 25°C (*P < 0.05). However, the PDI has been maintained within the acceptable range in both conditions. The zeta potential of LWS-SLN-Chit-Apt after 30 days of storage slightly increased in both temperatures. Moreover, the binding of the aptamer to LWS-SLN-Chit after the storage was validated through agarose gel electrophoresis (Figure 5). The absence of a band in the agarose gel electrophoresis for LWS-SLN-Chit-Apt indicated that the complex remains stable for 30 days at 0°C and 25°C.

**Table 4:** The particle size, zeta potential, and PDI of LWS-SLN-Chit-Apt after 30 days of storage at 4 and 25 °C.

**Temperature (°C)**	**Z-Average (nm)**	**Zeta Potential (mV)**	**PDI**
4 °C, Day 5	342 ± 22.4	+ 16.5 ± 2.3	0.22 ± 0.025
25 °C, Day 5	360 ± 28.1	+ 17.2 ± 2.8	0.24 ± 0.027
4 °C, Day 30	364 ± 18.9	+ 17.1 ± 1.6	0.26 ± 0.019
25 °C, Day 30	373 ± 20.4	+ 18.1 ± 2.1	0.29 ± 0.030

###  Drug Release 

**Figure 6:**
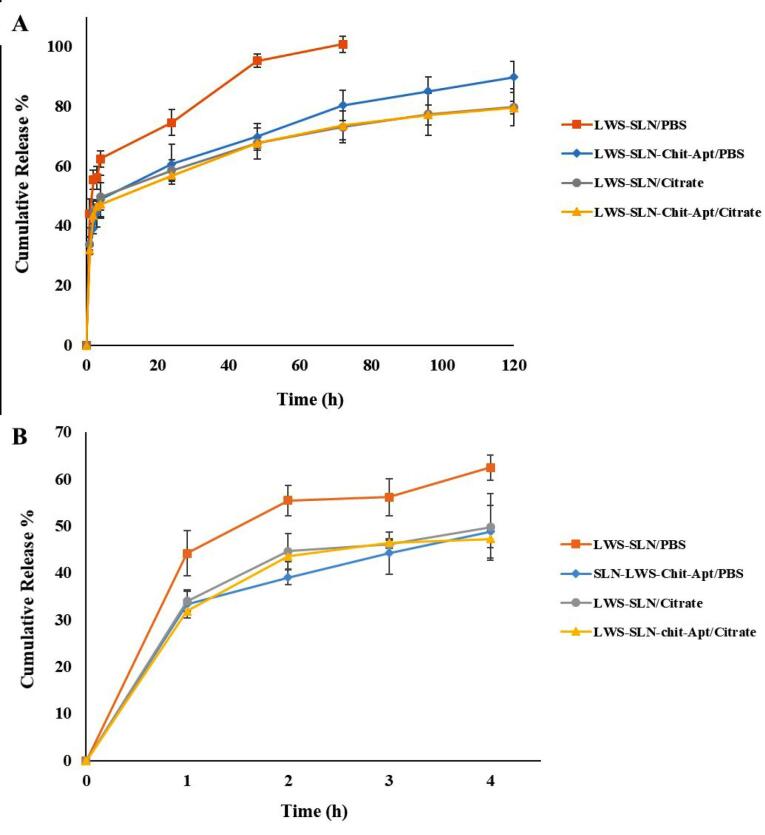
(A) Cumulative release of LWS-SLNs and LWS-SLN-Chit-Apt- in citrate buffer (pH = 5.5) and PBS (pH = 7.4) after 5 days. (B) is the onset of A and shows the first 4 h release profile of each sample

 The cumulative release profiles of LWS-SLNs and LWS-SLN-Chit-Apt in citrate buffer (pH 5.5) and PBS (pH 7.4) over 120 hours were represented in Figure 6. The release profile exhibited a biphasic release with an initial burst release in the early hours, followed by a slower, sustained release. After 4 hours, approximately 60% of LWS was released from LWS-SLNs in PBS, whereas LWS-SLN-Chit-Apt in PBS and both formulations in citrate buffer exhibited around 50% cumulative release. After 72 hours, 100% drug release was observed for LWS-SLNs in PBS, while approximately 85% was observed for LWS-SLN-Chit-Apt in PBS after 120 hours. However, the cumulative drug release in the acidic medium was similar for both formulations, with and without the aptamer, after 120 hours, reaching around 70%.

###  In Vitro Cytotoxicity 

**Figure 7:**
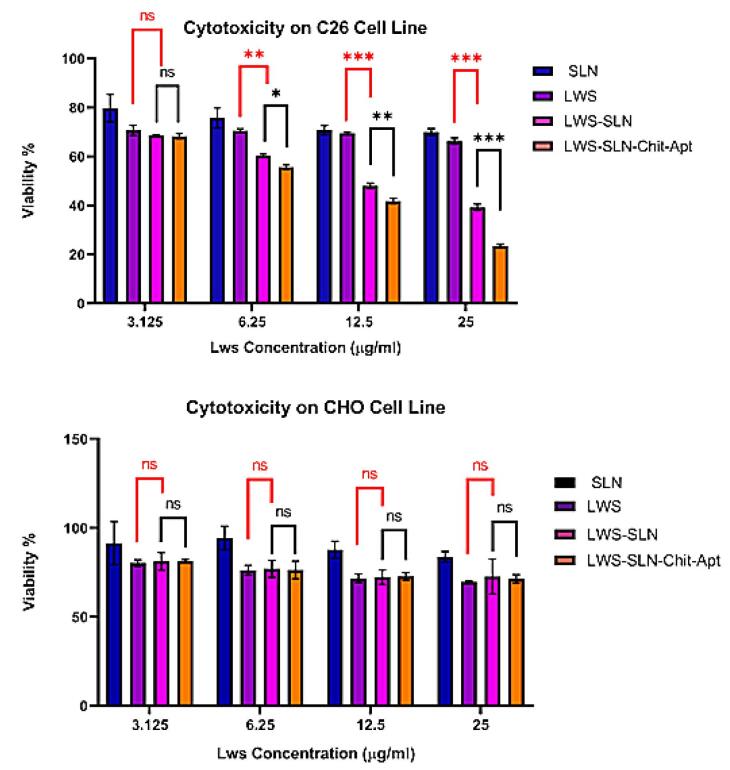
Dose-dependent inhibitory properties of SLNs, LWS, LWS-SLNs, and LWS-SLN-Chit-Apt against (A) C26 and (B) CHO cell viability after 24 h. (ns: not significant, *P < 0.05, **P < 0.01, ***P < 0.001)

 The MTT assay was performed to evaluate the viability of treated cells (C26 and CHO) with SLNs, LWS, LWS-SLNs, and LWS-SLN-Chit-Apt. As shown in Figure 7(A), bare SLNs exhibited approximately 20% inhibition of C26 cells. Increasing the concentration of free LWS from 3.125 to 25 µg/mL did not considerably change cell viability. In contrast, using SLNs as a nanocarrier (LWS-SLNs) led to a dose-dependent reduction in cell viability compared to free LWS. Notably, LWS-SLN-Chit-Apt demonstrated significant, dose-dependent inhibition of cell viability, particularly at LWS concentrations exceeding 3.125 µg/mL. As illustrated in Figure 7(B), the inhibitory effect of bare SLNs on CHO cells is lower than that observed in C26 cells, demonstrating the biocompatibility of SLNs components with normal cells. Furthermore, all LWS formulations exhibit a lower effect on the viability of the CHO cells when compared to the C26 cells. The inhibitory effect of LWS-SLNs and LWS-SLN-Chit-Apt remains relatively constant with the increasing of LWS concentration, showing consistent effects across all concentrations for both formulations on the CHO cells.

###  Cellular Uptake

**Figure 8:**
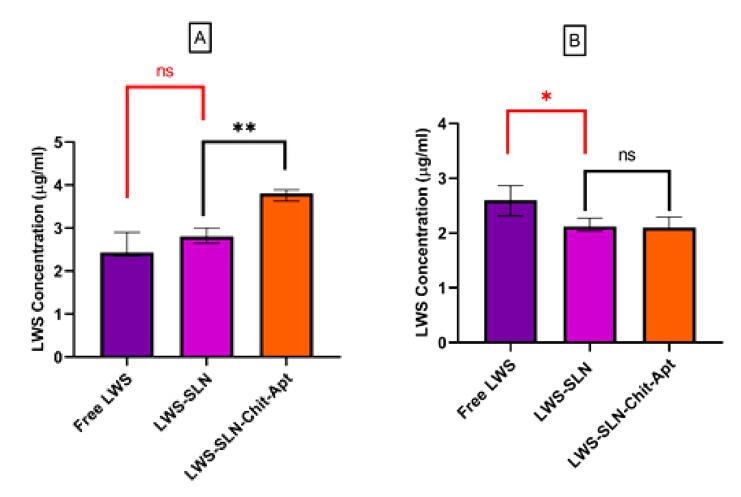
Cellular uptake of 10 µg/ml concentrations of LWS in different formulations (free LWS, LWS-SLNs, and LWS-SLN-Chit-Apt) for 4 hours by (A) C26 and (B) CHO cell lines (ns: not significant, *P < 0.05, **P < 0.01,***P < 0.001)

 The effectiveness of SLNs formulation and the aptamer conjugation in promoting cellular uptake of LWS was evaluated in C26 and CHO cells, with the results presented in Figure 8. The targeting of SLNs with the AS1411 aptamer significantly enhanced the cellular uptake in C26. However, in CHO cells, the uptake showed no significant differences with or without using the aptamer.

## Discussion

 Lawsone has confirmed significant efficacy in hindering the growth of various cancer cells. Its therapeutic benefits are notably enhanced when formulated with nanoparticles, which improve its bioavailability and enable targeted delivery. In the present study, chitosan-coated Lawsone-loaded solid lipid nanoparticles were fabricated and conjugated with the AS1411 aptamer to maximize their potential as a drug delivery system against colon cancer cells (C26).

 The type and percentage of lipids and surfactants are key parameters influence SLNs properties, including particle size, long-term stability, drug loading efficiency, and release behavior.^44^

 In SLNs preparation, approximately 0.1–30% (w/w) of solid lipid is dispersed in an aqueous phase, with surfactant concentrations ranging from 0.5 to 5% to enhance stability. ^45^ Similarly, in this study, the lipid and surfactant concentrations were selected within these ranges to optimize formulation outcomes. Precirol and Compritol have a partially amorphous, layered structure that gradually crystallizes over time. However, Compritol, which contains longer fatty acid chains than Precirol (behenate C22 versus palmitostearate C16–C18 esters), exhibits a slower crystallization rate, highly dependent on storage conditions, particularly temperature.^46^ Compritol was less effective than Precirol in producing small, homogeneous nanoparticles, and its use as a lipid matrix also resulted in gelation of the dispersion.^47^ The results of this study also indicate that SLNs formulated with Precirol as a lipid matrix produced particles with significantly smaller mean diameters than to those prepared with Compritol. Surfactants reduce nanoparticle size by lowering the surface tension between molecules.^48^ In this study, using Poloxamer 188 alone resulted in an unstable formulation, while Tween 80 alone produced a formulation with a low zeta potential. However, the combined use of Poloxamer 188 and Tween 80 resulted in the highest zeta potential, approximately -20 mV. In Rasouliyan et al. study, the combination of Tween 80 and Poloxamer 470 resulted in the optimal SLNs formation with the proper size and zeta potential (127 ± 3.1 nm and − 26.3 ± 0.22 mV).^20^ The main difference between our study and this article is that the targeting agent, AS1411 aptamer, was used in our study, which successfully targeted cancer cells while sparing normal cells, which has been well studied and verified in the cellular uptake assay. To achieve non-covalent conjugation of the aptamer to LWS-SLNs nanoparticles, the chitosan coating was performed. It means that the LWS-SLNs nanoparticles were coated with chitosan to form a positive charge on the particles for optimal electrostatic binding to the aptamer. Moreover, using sodium cholate as the co-surfactant in our study led to reducing particle size and enhancing the stability of nanoparticles. In addition, the amount of lawsone used in our formulation was much less than in other studies.

 The size, PDI, and zeta potential of SLNs are critical for their effectiveness as drug delivery systems. These parameters ensure physical and electrostatic stability, efficient drug loading, and enhanced cellular uptake capacity.^49,50^ Research indicated that SLNs with a particle size between 200 and 450 nm, a PDI of less than 0.3 are ideal for drug delivery.^51^ Also, Zeta potential is a key factor in determining nanoparticle stability, with an optimal range between -15 and -30 mV. Values outside this range indicate a higher likelihood of particle aggregation, compromising the stability of the formulation.^52^ The PDI represents the uniformity of nanoparticle size distribution, with values below 0.3 indicating a homogeneous SLNs dispersion. A lower PDI reflects better particle size uniformity, suggesting enhanced stability. Conversely, formulations with PDI values above 0.3 are more prone to aggregation and sedimentation over time, leading to reduced long-term stability.^52^

 So, according to Table 1, SLN-3 was the optimal formulation based on DLS results, exhibiting the smallest particle size among the formulations (259 ± 10.3 nm), along with an appropriate zeta potential (-20 ± 3.2 mV) and PDI (0.224 ± 0.02). The addition of sodium cholate as a co-surfactant in the SLN-3 formulation reduced the particle size by approximately 38% and resulted in a narrower size distribution (PDI = 0.12 ± 0.019). This demonstrates suitable particle size, and homogeneity, highlighting its potential for an effective nano delivery system. Using sodium cholate in preparing lipid nanocarriers offers several benefits, particularly in reducing particle size and enhancing stability. Previous studies using sodium cholate as a surfactant have demonstrated that it imparts desirable physical characteristics to the nanostructured lipid carrier, including a higher zeta potential, lower polydispersity index, and smaller particle size.^53^

 SEM image of LWS-SLNs showed that the synthesized particles are spherical with a smooth, uniform surface. The sphericity of SLNs offers several benefits, including favorable release profiles, tailored drug delivery, excellent physical-chemical stability, and the capability to deliver both hydrophilic and lipophilic drugs.^54,55^

 The FTIR result affirmed the chemical composition of the nanoparticle and the encapsulation of lawsone within the NPs. The first aromatic ring of lawsone is indicated by the wavelength 3378.92 cm^-1^, ascribed to the stretching vibration of the hydroxyl group.^56^ Additionally, the band at 1730.97 cm^-1^ in the LWS spectrum corresponds to the α-unsaturated carbonyl band 18. The SLNs spectrum showed major peaks at 2916.2 cm^-1^ and 2849.77 cm^-1^ due to C–H stretching, which is characteristic of Precirol®, the main component in SLNs.^37,57^ The presence of characteristic peaks in the LWS-SLNs spectrum confirms that each component remains in physical contact without undergoing chemical interaction. If a chemical interaction had occurred, it would result in the shifting or loss of prominent peaks. The absence of new peaks in the FTIR spectrum of LWS-SLNs indicates that no new molecular groups have been formed.^57^ DSC results reveal endothermic peaks at approximately 191.59°C, 54.51°C, and 56°C for LWS, SLNs, and LWS-SLNs, respectively. The observed endothermic peak for LWS aligns with other references that report its melting point in the range of 192–195°.^58^ The endothermic peak for SLNs showed a slight decrease compared to the melting points of Precirol® (65°C) and Poloxamer 188 (55°C).^37^ The thermogram of lyophilized LWS-SLNs did not display the endothermic peak for LWS around 190°C, suggesting that LWS was encapsulated in an amorphous or molecularly dispersed state within the NPs. These observations are consistent with findings from other studies.^37,59^

 Although the particle size increased during storage, especially at 37°C, LWS-SLNs remained stable, with DLS results for particle size, zeta potential, and PDI showing that all values remained within the acceptable range after long-term storage at various temperatures. Lower temperatures generally help maintain the NPs’ physical and chemical stability, whereas higher temperatures can accelerate degradation processes and destabilize them. Elevated temperatures increase the mobility of lipid molecules, leading to particle growth and changes in size distribution.^60^ This result aligns with findings from other study, where SLNs particle size enhanced quickly at raised temperatures but remained stable for over 180 days under refrigeration.^61^ In further research monitoring SLNs stability at 4–9 °C over four weeks, the PDI increased; however, mean particle size showed no significant change. ^54^ However, our results displayed that the PDI remained below 0.2 even after 6 months at 37°C.

 After coating LWS-SLNs with chitosan to create a positive charge for optimal aptamer binding, aptamer conjugation was performed. The increase in particle size to 350 ± 22.5 nm and the reduction in zeta potential to + 16 ± 2.1 mV indicate successful aptamer conjugation. This is consistent with other studies showing that aptamer conjugation alters zeta potential and increases particle size compared to formulations without the aptamer.^62^ Also, the stability of LWS-SLN-Chit-Apt was confirmed via DLS. The particle size and zeta potential before and after storage fall within the appropriate range for effective drug delivery. Additionally, Electrophoresis confirmed aptamer conjugation before and after storage, as the absence of the aptamer band in the gel for LWS-SLN-Chit-Apt indicated successful binding. This technique is commonly used to confirm aptamer binding, as demonstrated by Mansouri et al., who observed a similar absence of the AS1411aptamer band in the agarose gel electrophoresis of the vector-aptamer complex, suggesting successful conjugation of the aptamer to the vehicle. This absence indicated the effective binding, as unbound aptamers would otherwise appear as a distinct band.^63^

 The release profile demonstrated a biphasic release pattern. The rapid initial release is probably caused by LWS on the outer surface of the SLNs, whereas the sustained release is due to LWS encapsulated within the SLNs.^64^ A similar release pattern has been observed in another study comparing the release profiles of free 5-FU and 5-FU-loaded SLNs. This study showed that free 5-FU displayed a rapid release within just 6 hours. In contrast, the release of 5-FU from SLNs followed a biphasic pattern, with an initial burst release of approximately 40-45% within the first 3 hours, followed by sustained release over 48 hours.^37^ The capacity to modulate drug release in response to pH variations can significantly enhance therapeutic efficacy, especially for targeting tumor microenvironments, which tend to be more acidic (pH ≈ 6) than normal tissues and the bloodstream (pH ≈ 7.4).^65^ In this study, the nanoparticles were coated with chitosan to create a positive charge on the particles for optimal aptamer binding. In other words, to achieve non-covalent conjugation of the aptamer to NPs (LWS-SLNs), chitosan coating was performed. Chitosan contains free amino groups in its structure with a pKa of 6.3. At low pH, the primary amino groups are protonated, leading to electrostatic repulsion between the monomeric units resulting in enhanced chitosan solubility in low pH. In tumor environments, pH is about 5.5 and can incite the release of drugs that are loaded in the nanoparticles.^66^ As a result, at this pH, the behavior of nanoparticles with chitosan coating was similar to that of nanoparticles without chitosan coating.

 However, the AS1411 aptamer conjugation to the surface of LWS-SLNs reduced drug release in PBS, indicating a lower release rate in the bloodstream. This suggests an improved therapeutic effect by decreasing drug release in circulation and potentially increasing drug accumulation at the target site.

 Numerous studies have verified the anticancer properties of lawsone, with various underlying mechanisms proposed. Majiene et al. stated that lawsone reduces the viability of C6 glioblastoma cancer cell lines and exhibits robust antioxidant activity.^67^ Similarly, another study displayed that lawsone derivatives induce apoptosis and decrease the migratory capacity of melanoma cells.^68^ Lawsone and its derivatives have also been shown to activate apoptosis by upregulating the expression of the pro-apoptotic gene BAX and downregulating the expression of the anti-apoptotic gene BCL2.^18,69^ Consistent with these findings, the present study revealed that LWS-SLN-Chit-Apt effectively suppressed C26 cell growth, while maintaining the viability of normal CHO cells. Increasing free LWS concentration had minimal impact on cell viability due to limited cell availability. In contrast, using SLNs as a nanocarrier (LWS-SLNs) made a dose-dependent decrease in cell viability, likely because of enhanced cellular uptake and controlled release of the therapeutic agent. The IC_50_ of LWS-SLNs was 15 µg/mL, which is 5-fold lower than free LWS’s. This finding is supported by another study, which demonstrated that encapsulating LWS in nanoniosomes could significantly enhance the anti-tumor effect of the formulation in MCF-7 cell lines compared to free LWS. ^19^ In addition, the IC_50_ for LWS-SLN-Chit-Apt after 24 hours was 10.36 µg/mL. This is most likely related to the AS1411 aptamer targeting, which particularly binds to the highly expressed nucleolin on the plasma membrane of cancer cells. Nucleolin, in normal cells is presented in the nucleus whereas in cancer cells, it is located also in the cytoplasm and at the cell surface. Nucleolin, presents on the cell surface, exhibits an important role in the absorption and signaling of different growth factors which support the cancer cell proliferation. Vice versa, nucleolin located in the cytoplasm improves the anti-apoptotic mRNAs and miRNAs levels, contributing to the survival of cancer cells. So, the non-nuclear nucleolin represent highly selective targets for cancer treatment, as they mostly exist in cancer cells rather than in normal cells. Moreover, these forms have substantial roles for the existence of cancer cells that are not critical for normal cells.^62^ In various investigations has been shown that AS1411 aptamer could efficiently interact with nucleolin so prevent proliferation and induce cell death in different types of cancer cell lines. When the interaction of nucleolin with its targets is disrupted, then, DNA replication interrupted, as well as, S-phase arrest and stabilizing the mRNA of B-cell lymphoma 2 (BCL-2) is occured.^70^

 Wan et al. reviewed the various studies that have thoroughly investigated how the AS1411 aptamer interacts with nucleolin and enters the cells.^71^ For example, Reyes-Reyes et al. investigated the different mechanisms of AS1411 uptake in cancer cells and non-malignant cells. Their study showed that macropinocytosis is the main mechanism in cancer cells for AS1411 uptake, whereas clathrin- or caveolae-dependent rout is used in non-malignant cells.^72^ In another study, different caveolin-dependent endocytosis inhibitors such as Genistein and methyl-β-cyclodextrin and clathrin-dependent endocytosis inhibitors including sucrose and monodansylcadaverine were used to explore the AS1411 aptamer internalization mechanism in breast cancer stem (BCS). In both situation, the aptamer could enter the BCS cells efficiently, while the aptamer internalization was blocked in the differentiated breast cancer cells.^73,71^ Another study also indicated that the AS1411 aptamer is taken up through a nucleolin-dependent mechanism by macropinocytosis pathways.^26^ Other studies have confirmed that NPs functionalized with the AS1411 aptamer demonstrate increased cellular uptake and cytotoxicity in nucleolin-expressing cancer cells, resulting in elevated intracellular ROS levels and the suppression of tumor growth. ^30,63^ Also, in both *in vitro* and *in vivo* studies, the presence of the aptamer on the surface of drug carrier, nanobubbles, expressively enhanced the cellular uptake and cytotoxicity in C26 cells compared to carriers without the aptamer. Specifically, the system without the aptamer demonstrated 58% tumor growth suppression, whereas the aptamer-conjugated system achieved 95% tumor growth suppression.^74^ Similarly, the conjugation of an aptamer to doxorubicin-containing SLNs exhibited a greater inhibition of cell proliferation in the MDA-MB-468 cell line than the formulation without the aptamer.^75^ In this study, all LWS formulations had minimal impact on CHO cell viability. This can be attributed to the fact that targeting nanoparticles with the AS1411 aptamer does not significantly affect the cell affinity for low nucleolin-expressing cell lines, such as CHO.^30^ Also, targeting SLNs with the AS1411 aptamer significantly increased cellular uptake in C26 cells, while no substantial difference was shown in CHO cells. This suggests that using the AS1411 aptamer for detecting cancer markers like nucleolin, can effectively facilitate the NPs transfering to the cytoplasm in nucleolin-overexpressing cells, resulting in enhanced uptake in these cells.^76^ Similar outcomes have been stated in other studies, where AS1411 aptamer-functionalized NPs demonstrated improved cellular uptake and efficacy in MCF7 cells compared to non-targeting NPs, because of the aptamer’s high affinity for the overexpressed nucleolin on the MCF7 cell surface.^77^ Additionally, when AS1411-aptamer was used on the surface of drug-loaded NPs, the cellular uptake in nucleolin-positive cell lines (A375 and C26) was increased, and more cytotoxicity was achieved compared to nucleolin-negative L929 fibroblasts.^63^

 The cellular uptake assay was conducted at a concentration equal to the IC₅₀ of the targeted complex (LWS-SLN-Chit-Apt), which represented a therapeutically relevant dose, where the biological effect (cytotoxicity) was directly linked to the internalization efficiency. This approach permits a comparative evaluation of the targeting efficacy of the aptamer-functionalized nanoparticles against non-targeted controls, LWS-SLNs and free drug in nucleolin-positive cells (C26 cells) and nucleolin-negative cells (CHO cells). This attitude has been frequently used in previous studies for instance as performed in Abdollahzade et al.^78^ or Choi et al. investigations.^79^

 When evaluating cytotoxicity, it is necessary to highlight that bare SLNs inhibited C26 cell viability by ~20%. It has been shown that the type of the lipids and their concentrations in SLNs formulation had a direct influence on the cytotoxicity. Furthermore, SLNs’ cytotoxicity was probably a result of some products of enzymatic degradation of SLNs including free fatty acid or may be the side effects of surfactants such as Tween80.^80^ For example, in Guo et al.’s study, bare SLN showed cell survival of about 80% on different cells, whereas drug-loaded formulations could be more cytotoxic. The authors attributed this cytotoxic effect of bare SLN mainly to excipient/surfactant interactions at the cell membrane rather than to the solid lipid core itself.^81^ Another study indicated that the cytotoxicity of blank SLNs may be ascribed to the side effects of surfactants used in their formulations.^82,83^ Recently, Moraes-Lacerda et al. reviewed the literatures and critically investigated the biocompatibility and cytotoxicity of empty SLNs. In this article, the relationship between SLNs and cells, the activation of signaling pathways and their effects, as well as minor alterations and cytotoxicity, have been carefully discussed. Ultimately, they concluded that the cytotoxicity of SLNs could not be ignored.^84^ Considering this information is important for developing the subsequent SLNs formulations and their translation into clinical applications and future products.

 Nevertheless, some limitations should be considered. For instance, ultrasonication generates SLNs without requiring organic solvents and present a fast, simple, and efficient method. However, it has some drawbacks due to the metal impurities production, probable physical instability and particle growth during storage. For scaling up this technique, the frequency and strength of ultrasonication would be adjusted to regulate the size of nanoparticles.^85^ For clinical application, some other points should be noted and investigated more including potential off-target effects of AS1411, the appropriate storage procedures and the long-term stability particularly due to the presence of the aptamer and final validation through *in vivo* experiments and clinical studies.

 However, due to the appropriate characteristics of the AS1411 aptamer, it is one of the most widely investigated aptamers and has been used in clinical studies. It has proceeded to phase II clinical trials targeting acute myeloid leukemia and renal cell carcinoma.^86,87^ A randomized Phase II trial considered the efficacy of the AS1411 aptamer in combination with cytarabine in patients with relapsed or refractory acute myeloid leukemia (AML). The outcomes indicated that anti-leukemic effects were improved while maintaining an acceptable safety profile compared to historical controls, representing the necessity for further investigation of AS1411 as a chemo-sensitizer in blood cancers. ^86^ Another clinical trial related to evaluating AS1411 effects in patients with metastatic clear cell renal cell carcinoma (RCC) who had not responded to tyrosine kinase inhibitor treatment through a phase II single-arm study. Thirty-five patients participated in this trial and received the treatment. The notable 84% reduction in tumor burden based on RECIST 1.0 criteria was reported in one patient, which was long-lasting about two years after treatment. Additionally, an adverse event associated with the AS1411 aptamer occurred in 34% of patients, which was classified as mild or moderate.^87^

## Conclusion

 In this study, we successfully prepared SLNs containing Lawson, targeted with the AS1411 aptamer, to evaluate their anticancer efficacy against colon cancer cells (C26). The LWS-SLNs were fabricated using high-shear homogenization and ultrasound methods, resulting in NPs with appropriate particle size, surface charge, and encapsulation efficiency. Our cellular studies demonstrated targeted drug delivery to nucleolin-positive cancer cells, leading to higher cellular uptake and superior cytotoxicity in these cells. Our findings propose that the LWS-SLN-Chit-Apt is an encouraging drug delivery system for improving the bioavailability of lawsone and enhancing its beneficial effects against cancerous cells.

 For future perspectives, the study of the dose-dependent cellular uptake kinetics for prepared formulations will be a focus of the next research. Moreover, the present study demonstrated the promising results for the stability of aptamer-functionalized nanoparticles over a month, but a long-term stability investigation (e.g., over 6-12 months) under ICH-guided conditions is an indispensable next step. The future work will include not only dose-dependent cellular uptake kinetics but also an evaluation of aptamer stability over time, which is necessary for clinical translation. Additionally, nucleolin knockdown studies will validate the mechanistic features of AS1411 uptake in our subsequent investigations.

## Competing Interests

 The authors declare no conflict of interest.

## Ethical Approval

 Not applicable.
